# Cross-Cultural Validation of Manual and Automated Methods for Extracting Trauma Memory Features and Predicting PTSD in Young Populations

**DOI:** 10.1007/s10802-026-01469-4

**Published:** 2026-06-11

**Authors:** Alessandra Giuliani, Tamsin Sharp, Yeukai Chideya, Katie Spurgin, Richard Meiser-Stedman, Mark Tomlinson, Sarah L. Halligan

**Affiliations:** 1 Department of Psychology, University of Bath, Bath, BA UK; 2 Bristol Medical School, University Of Bristol, Bristol, UK; 3 Institute for Life Course Health Research, Department of Global Health, Stellenbosch University, Stellenbosch, South Africa; 4 Department of Psychology, Institute of Psychiatry, Psychology and Neuroscience, King’s College London, London, UK; 5 School of Nursing and Midwifery, Queen’s University Belfast, Belfast, UK

**Keywords:** Post-traumatic stress, Trauma memory, Child and adolescent, Cross-cultural

## Abstract

Cognitive models of post-traumatic stress disorder (PTSD) propose that traumatic events are encoded in fragmented, sensory-rich, and poorly contextualised memories that maintain PTSD symptoms. Although this hypothesis has received support in adults, evidence in children and adolescents remains limited, methodologically heterogeneous, and largely restricted to high-income, English-speaking settings. We pooled data from 407 children and adolescents exposed to trauma (6–17 years) recruited from emergency departments in South Africa and the United Kingdom. A month after their traumatic experience, participants were asked to recall their traumatic event and completed self-report measures of trauma memory quality and PTSD symptoms. Trauma narratives were transcribed, and indices of coherence and sensory valence were derived using three narrative-based approaches. We examined the validity of narrative-derived indices with self-reported memory quality, their associations with concurrent PTSD symptoms, and whether these patterns varied across cultural context and age. Across both cultural contexts, self-reported trauma memory qualities were significantly associated with PTSD symptoms. By contrast, narrative-derived indices showed no convergence with the corresponding self-report measures and did not meaningfully predict PTSD symptoms. Limited age-related differences were observed: in the South African sample, self-reported trauma memory characteristics were associated with PTSD only among older youth, whereas in the UK sample, they were predictive across age groups. In children and adolescents exposed to trauma, commonly used narrative-based indices of memory disorganisation and sensory content have no construct validity nor clinical utility for predicting PTSD. In contrast, self-report measures of trauma memory features emerge as a reliable and cross-culturally valid indicator of PTSD symptomatology.

**Supplementary Information:**

The online version contains supplementary material available at 10.1007/s10802-026-01469-4.

## Introduction

Many children experience traumatic events, such as serious accidents, interpersonal violence, or acute medical episodes (Lewis et al., 2019). For most, post-traumatic reactions are transient (Hiller et al., 2016). However, a minority go on to develop persistent post-traumatic stress symptoms (PTSS), including re-experiencing, avoidance, hyperarousal, and negative alterations in cognition and mood, that may meet diagnostic criteria for post-traumatic stress disorder (PTSD) in approximately 15% of cases (Alisic et al., 2014; American Psychiatric Association, 2013). Early identification of children at heightened risk for developing PTSD is critical for guiding timely and targeted interventions, and psychological theories can provide valuable insights into the factors associated with the emergence of post-traumatic psychopathology.

Cognitive models of PTSD highlight trauma memory characteristics as central to symptom development and maintenance (Brewin et al., 1996; Ehlers & Clark, 2000). According to these models, trauma memories are encoded and stored differently from typical autobiographical memories, exhibiting greater fragmentation, sensory richness, and contextual disconnection (Brewin et al., 1996; Ehlers & Clark, 2000). These theories have been primarily developed and tested in adult populations. However, evidence suggests that similar aetiological factors are relevant in youth, supporting the application of these theoretical frameworks to children and adolescents (Reed et al., 2023). However, when specifically examining trauma memory characteristics, developmental differences in children’s language and autobiographical memory skills may confound measurement (Fivush, 2011). It is unclear whether observable features of how children recount their trauma can meaningfully index memory processes or predict PTSD symptomatology. It is therefore critical to evaluate the suitability of different trauma memory assessment methodologies in younger populations and to determine their relevance for identifying children at elevated clinical risk.

### Measuring Trauma Memory Characteristics

A range of methodologies have been developed to assess trauma memory characteristics, resulting in inconsistencies across studies and limited comparability. Broadly, these methods fall into two categories: self-report questionnaires and narrative-based approaches.

### Self-Report Measures of Trauma Memory

The most widely used self-report measures in PTSD research are the Trauma Memory Quality Questionnaire (TMQQ; Meiser-Stedman et al., 2007) and its Adapted Child version (ACTMQQ; Hiller et al., 2021). These scales index children’s own perceptions of how disorganised or sensory-rich their trauma memories are. Given that these memory qualities are inherently subjective, these self-report measures allow direct insight into children’s appraisals of trauma memories. That is, they provide access to internal, phenomenological qualities of memory which are not easily observable. From a cognitive perspective, such appraisals may reflect how accessible, intrusive, or poorly contextualised the memory feels to the individual, processes that are central to leading cognitive models of PTSD (Brewin et al., 1996; Ehlers & Clark, 2000). Across multiple studies, self-reported trauma memory qualities have shown moderate to strong associations with concurrent PTSD symptoms (Reed et al., 2023). This suggests that children’s subjective appraisals of their memories may capture clinically relevant aspects of memory processing that are closely related to symptom development. Self-report measures may also be less affected by developmental differences in expressive language ability compared to narrative-based approaches, making them potentially more suitable for assessing trauma memory characteristics in younger populations.

### Narrative-Based Approaches

Narrative methods, in contrast, focus on the observable structure and content of an oral or written trauma account. Compared to self-report measures, narrative methods offer the advantage of capturing more spontaneous and nuanced memory expressions, potentially reflecting the natural structure and organisation of the memory more objectively. However, it has been argued that the extent of disclosure may differ between age groups and trauma type (McElvaney et al., 2014). Moreover, narrative methodologies require validation in children, as children tend to show poor integration of autobiographical memories because of less-developed language abilities (Fivush, 2011; Nelson & Fivush, 2004). Specifically, three main narrative-based approaches have been employed in the literature: manual coding, word-count-based methods, and Natural Language Processing (NLP).

#### Manual Coding

A common method for extracting memory characteristics from trauma narratives is manual coding, in which trained raters code trauma narratives for features such as repetition, disorganised or unfinished thoughts, and sensory references (Foa et al., 1995; Halligan et al., 2003; Salmond et al., 2011). This approach allows for nuanced, context-sensitive judgements. However, it is resource-intensive, vulnerable to rater subjectivity, and has produced mixed findings in youth: while some studies have found no significant associations between narrative disorganisation or sensory detail and PTSS (e.g., McKinnon et al., 2017), others have reported modest positive relations for specific narrative features only (e.g., McGuire et al., 2021), even when similar coding schemes have been used.

#### Word-Count-Based Methods

Word-count-based methods quantify the presence of particular linguistic categories within a narrative by counting words belonging to predefined dictionaries or semantic classes. A widely used dictionary-based text analysis tool is the Linguistic Inquiry and Word Count (LIWC; Pennebaker et al., 2015), which applies a validated dictionary to text to identify linguistic and psychological features, producing scores that represent the percentage of words falling into relevant categories. In adult trauma research, LIWC indices such as cognitive mechanism words (e.g., “because”) or analytic thinking have been interpreted as indicators of more coherent and organised trauma memories (Jelinek et al., 2010) and have shown associations with PTSD severity (Jaeger et al., 2014; Kleim et al., 2018). However, evidence in children and adolescents is far more limited and notably inconsistent. For example, Miragoli et al. (2019) reported associations between LIWC-derived emotional language and children’s trauma responses following an earthquake, whereas other studies of youth have failed to identify reliable links between LIWC indices and PTSD symptoms or narrative organisation. Importantly, there is little evidence that LIWC captures structural features such as fragmentation or disorganisation in young populations, raising concerns about its sensitivity to the trauma memory properties emphasised in cognitive models of PTSD. Moreover, because LIWC does not account for linguistic context or higher-order narrative structure, its applicability may be particularly constrained where children’s developing language abilities limit the complexity and coherence of trauma recall.

#### Natural Language Processing (NLP)

NLP approaches offer a more sophisticated alternative to word-count-based approaches by analysing the relationships between words and ideas across the entire narrative. Techniques such as Latent Semantic Analysis, Latent Dirichlet Allocation, and computational coherence algorithms learn from large text corpora and can quantify semantic relatedness, topic structure, and global coherence without relying on predefined dictionaries (Landauer et al., 1998; Li, 2024). Early work suggests that NLP-derived features can classify PTSD status from adult trauma narratives with promising accuracy (Bartal et al., 2022; Zhang, 2022). However, NLP tools are typically trained on English-language data from adult populations (Hovy & Prabhumoye, 2021), and their performance with child narratives, particularly in non-English or translated narratives, remains unexplored. This limitation reflects broader structural biases in NLP development, whereby only a small subset of the world’s languages are sufficiently resourced to support robust model training (Blasi et al., 2022; Joshi et al., 2020; Udeze, 2025). Crucially, these challenges extend beyond cross-language differences. Even within English, substantial sociolinguistic and cultural variation exists, including differences in dialect, narrative style, and culturally shaped modes of emotional expression. Such variation can influence how experiences are encoded in language, yet is often underrepresented in the standardised corpora used to train NLP models (Zampieri et al., 2020). As a result, models developed on dominant or “standard” forms of English may inadequately capture the linguistic features of narratives produced in diverse cultural contexts.

### Current Gaps

In sum, both narrative-based and self-report approaches offer distinct strengths and limitations. However, several important gaps remain in the trauma narrative literature. First, the majority of work examining trauma memory characteristics has been conducted with adults, and the limited research involving children and adolescents has typically treated them as a homogeneous group (McGuire et al., 2021). Yet, substantial developmental changes in language, autobiographical reasoning, and metacognitive abilities occur across middle childhood and adolescence (Fivush, 2011; Nelson & Fivush, 2004). These evolving capacities affect both how trauma memories are encoded and how they can be expressed, meaning that methods appropriate for older adolescents may be poorly suited to younger children and vice versa. Second, existing work on trauma memory characteristics in youth has been conducted almost exclusively in high-income, English-speaking contexts, despite PTSD being a global condition (Atwoli et al., 2013; Figueira et al., 2007; Pham et al., 2004). It is unclear whether narrative-based indices derived from English-language tools (e.g., LIWC dictionaries, NLP models) perform adequately when applied to narratives translated from other languages. Different languages and cultural contexts may exhibit distinct structural and lexical patterns (Schnell & Schiborr, 2022), which may affect how we interpret indices such as ‘cognitive mechanism words’ or ‘analytic thinking’. Finally, very few studies have directly compared multiple memory measures, manual coding, automated narrative indices, and self-report within the same sample. Direct comparisons are essential because they allow assessment of convergent and discriminant validity across methods, clarifying whether different tools are capturing the same underlying memory constructs or instead reflecting distinct processes such as language ability or subjective appraisal. One notable exception is Rubin and colleagues (2016), who reported the predictive value of narrative features in adults. However, that study focused on a small sample and did not include children or adolescents, limiting its relevance for developmental applications.

### Current Study

The current study addresses these gaps by providing a systematic, cross-cultural comparison of manual coding, word-count and NLP approaches, and self-report questionnaires for assessing trauma memory characteristics in children and adolescents. These methods are evaluated across two large samples of youth exposed to trauma: one from a low- and middle-income country (LMIC) context (South Africa) and the other from two studies conducted in the United Kingdom, a high-income country (HIC). Cultural factors, such as willingness to discuss traumatic experiences and awareness of mental health difficulties, may well differ between these samples (Monnapula-Mazabane & Petersen, 2023). For example, UK contexts often place greater emphasis on individual emotional awareness and verbal expression of internal states, which may facilitate more elaborated narratives and higher endorsement of subjective memory qualities in self-report measures. In contrast, in some South African contexts, cultural norms may place greater emphasis on behavioural coping, social resilience, or reduced verbal disclosure of distress, which could result in less elaborated narratives or lower endorsement of internal states despite similar underlying experiences (Monnapula-Mazabane & Petersen, 2023). By comparing findings across these settings, the study examines the extent to which cultural factors influence the validity of each approach and their capacity to identify children at heightened risk of PTSD. We operationalise validity as the correspondence between narrative-based and self-report indicators of memory qualities and predictive utility, reflected in associations with post-traumatic stress symptoms, and we examine developmental differences by comparing younger (6–12 years) and older (12–17 years) participants.

Specifically, the study aims to:

1. Evaluate the validity of manual coding, word-count, NLP, and self-report methodologies for assessing trauma memory characteristics in children across different cultural and language contexts.
2. Assess the extent to which memory characteristics derived from each method predict concurrent PTSD symptom severity.
3. Examine whether the predictive utility of these methods differs between younger and older children.

## Methods

### Participants

This study pooled secondary data from three closely aligned cohorts of children and adolescents who had been exposed to trauma, recruited from hospital emergency departments after a traumatic incident. In total, the combined dataset comprises 407 participants from South Africa and the United Kingdom (UK). The South African sample was drawn from the Sinethemba study (Sharp et al., 2024), a longitudinal project investigating post-trauma adaptation among youth living in socioeconomically disadvantaged communities in Cape Town. Participants (*N* = 222; 154 male participants; 61.6%) were aged 8–17 years (M = 12.41, SD = 1.29) and were all identified as Black African. Caregiver characteristics were as follows: caregivers (M = 41.16, SD = 10.96) were predominantly mothers (68.0%), followed by other biological relatives such as grandparents or uncles (20.1%), fathers (4.7%), stepmothers (3.0%), other caregivers (1.7%), and sisters (1.3%). Regarding marital status, 54.6% were married or in a relationship. Caregivers’ educational attainment was predominantly at the school level: 88% had completed up to Grade 12, 11% held a post-matric certificate or diploma, and 1% held a degree.

The UK sample consisted of data from two studies: PROTECT (*N* = 128; Hiller et al., 2018) and ASPECTS (*N* = 57; Meiser-Stedman et al., 2019), yielding a combined sample of 185 children and adolescents exposed to trauma (118 male participants; 63.8%) from the UK, aged 6–17 years (M = 11.78, SD = 1.17). The UK sample was predominantly White (93.8%). Caregiver characteristics for the UK sample included a mean caregiver age of 39.7 years (SD = 7.0), with 90% being mothers. The majority were married or cohabiting (74%), and educational attainment was distributed across school-level qualifications (27%), further education (38%), and higher education (35%).

Across cohorts, trauma exposure differed by sample. In the UK sample, trauma types included road traffic accidents (49.4%), accidental injuries (31.5%), assault (6.7%), acute medical emergencies (5.6%), dog bites (2.2%), and other events (7.9%). In the South African sample, trauma exposure comprised accidental injuries (39.2%), road traffic accidents (24.8%), assault (14.4%), other events (18.5%), and acute medical emergencies (0.5%).

### Procedure

Across studies, procedures followed similar protocols. Detailed recruitment procedures and samples are described in the original publications of their studies. Across all studies, written informed consent was obtained from caregivers, and age-appropriate assent from all child and adolescent participants was obtained at the point of recruitment. Trained research staff explained the study aims, procedures, confidentiality, and the voluntary nature of participation to both caregivers and participants, ensuring comprehension and providing opportunities to ask questions. Participants were informed that they could withdraw at any time without consequence. At any follow-up assessments, consent and assent were reconfirmed prior to data collection. Approximately a month following the traumatic event, participants and their caregivers completed an interview and a battery of self-report measures. During the interviews, participants were asked to recall their traumatic incident using the following question: “I would now like you to tell me about the event that led to your recent hospital visit in as much detail as you can. You can do this in any way that you want; there are no rules. Try to tell me everything you know about what happened and include anything about what has happened since that you think is important. I will leave you to talk for as long as you like”. Prompts (e.g., “Do you remember any more?”) were given by the interviewer when the child disclosed little information.

All interviews were audio-recorded. For the South African cohort, interviews were conducted in isiXhosa or English, depending on the participant’s preference. For the UK cohort, interviews were conducted in English. Audio recordings were transcribed verbatim by trained transcribers. For the South African cohort, isiXhosa narratives were translated into English, prior to analysis, following a multi-step procedure designed to preserve meaning and minimise semantic loss. This included forward translation by bilingual researchers, review and discussion within the research team, and resolution of discrepancies through consensus. All subsequent narrative coding and automated text analyses were conducted on the English-language transcripts.

#### Narrative Pre-Processing

Before applying manual and automated methods for trauma memory feature extraction, narrative transcripts underwent standardised pre-processing to ensure consistency across studies. Narratives containing fewer than 50 words (2 in the South African Sample and 1 in the UK sample) were excluded, consistent with prior methodological conventions, which indicate that very short texts provide insufficient linguistic content for reliable coding and computational analysis (Pennebaker et al., 2015). Transcripts were then cleaned to retain only the participants’ speech and remove interviewers’ questions and comments. Participants’ responses to specific follow-up questions were excluded to preserve the narrative’s natural flow and avoid artificially inflating coherence scores. No further pre-processing was performed (e.g., stop-word removal, lemmatisation, or stemming) in order to preserve the original linguistic properties of the narratives (Khurana et al., 2023).

### Materials

Demographic information, trauma-related details (including type of event and basic injury characteristics), and self-reported measures of trauma memory quality and PTSD symptoms were obtained from children and adolescents during the interview. For the South Africa sample, questionnaires were translated into isiXhosa prior to administration. Translation followed established forward–backward translation procedures. Materials were first translated from English into isiXhosa by bilingual research staff and then independently back-translated into English by a separate bilingual speaker. Discrepancies were reviewed and resolved through discussion within the research team to ensure conceptual equivalence.

#### PTSD Symptoms

PTSD severity was measured using established age-appropriate clinical measures of PTSD. Specifically, the PROTECT study used the University of California at Los Angeles Reaction Index (UCLA-RI; Steinberg et al., 2013). The ASPECT and Sinethemba studies used the Children’s PTSD Symptom Scale Self-Report *(*CPSS-5-SR; Foa et al., 2018). Both instruments assess the frequency and severity of PTSD symptoms over the past month. The UCLA-RI yields total scores ranging from 0 to 80, whereas the CPSS-5-SR yields total scores ranging from 0 to 60. To account for this, total scores from both scales were divided by their respective maximums, yielding standardised scores ranging from 0 to 1.

#### Self-Reported Trauma Memory Quality

Self-reported trauma memory characteristics were assessed using the Trauma Memory Quality Questionnaire (TMQQ; Meiser-Stedman et al., 2007) in the ASPECTS cohort and the Adapted Child Trauma Memory Quality Questionnaire (ACTMQQ; Hiller et al., 2021) in the PROTECT and South African cohorts. The TMQQ comprises 11 items that assess two theoretically derived dimensions of trauma memory: disorganisation (e.g., confusion, gaps, difficulty piecing the memory together) and sensory qualities (e.g., vivid perceptual details). The ACTMQQ retains all TMQQ items and includes an additional seven items to broaden developmental suitability and enhance coverage of both domains, while maintaining the same two-factor structure.

To ensure direct comparability across cohorts, only the 11 items common to both versions were included in the present analyses. Each item is rated on a 4-point Likert scale (1 = strongly disagree; 4 = strongly agree). Subscale scores for disorganisation and sensory qualities were calculated as the mean of their respective items, generating scores from 1 to 4, with higher values indicating more fragmented and more sensory-vivid trauma memories.

#### Manual Coding

Manual coding produced three variables: a global disorganisation rating, an elements-of-disorganisation score, and a sensory score. It was carried out by three trained raters using a coding scheme adapted from Foa et al. (1995) and previously employed by McGuire et al. (2021). The manual scheme is available in the supplementary materials (Material A). Raters were blind to the study aim, PTSD severity, and participants’ demographics, and each rater coded all narratives. To minimise coder drift (Myford & Wolfe, 2009), the coding team held regular calibration meetings throughout the coding period and jointly reviewed a subset of narratives at predefined intervals. Inter-rater reliability was assessed using the intraclass correlation coefficient (ICC), based on a mean-rating (K = 3), two-way random-effects model. Across coding categories, reliability was good to excellent, with ICC values ranging from 0.76 to 0.95. Discrepancies were resolved through consensus discussion; where consensus could not be reached, disagreements were resolved by an independent third-party reviewer, which was required for approximately 10% of coded instances, consistent with best-practice guidelines (Syed & Nelson, 2015).

Following the manual, narratives were divided into chunks coded as repetitions, organised thoughts, disorganised thoughts, unfinished thoughts, pain utterances, and sensations. Based on the manual instructions, raters also provided a global rating score (0–10) for the degree of memory disorganisation of the narrative as a whole, with higher scores indicating greater disorganisation. Repetition, organised thoughts (reversed), disorganised thoughts, and unfinished thoughts were transformed into z-scores and combined to comprise ‘elements of disorganisation’. Pain and sensory categories were transformed into z-scores and combined to produce the ‘sensory’ variable.

#### LIWC

Linguistic Inquiry Word Count is used to produce the two outcome variables of Disorganisation and Sensory. The Disorganisation variable was computed as the composite score for Cognitive Processes (the percentage of words related to the category containing 797 words, such as “cause”, “know”, and “ought”) and Analytical Thinking. Analytical Thinking is a summary measure representing the degree to which the language in the text suggests formal, logical, and hierarchical thinking patterns. The Sensory variable was computed through the Perception category (sum of the percentage of terms related to “sight”, “hearing”, and “touch”/ “feeling”).

#### NLP

Several metrics of overall text semantic overlap extracted using TAACO (Tool for the Automatic Analysis of Cohesion; Crossley et al., 2016), a free automatic text analysis software, were aggregated to compute a disorganisation score. TAACO computes semantic overlap (lexical cohesion within a text) using three computational models: latent semantic analysis (LSA) (Landauer et al., 1998), Latent Dirichlet Allocation (LDA) (Blei et al., 2003), and Word2vec (Mikolov et al., 2013). All three scores range between 0 and 1. LSA and Word2vec are computed using the cosine similarity between segments. LDA scores are calculated from the Jensen-Shannon divergence between the normalised combined vector weights of words within each segment. Therefore, the LDA score indicates dissimilarity between adjacent segments and was reversed. Each narrative’s similarity score was computed by averaging the three measures. Each narrative was assigned a final score ranging from 0 (low cohesion) to 1 (high cohesion). Aggregating text cohesion metrics has been shown to capture text cohesion reliably (Miani et al., 2022).

We employed the concreteness norms from Brysbaert et al. (2014) to capture the narratives’ sensory content. This is a norms document for 40,000 English lemmas, scored on a concreteness scale from 1 to 5. More concrete words are easier to imagine, visualise, and experience; hence, a concreteness score can be utilised to capture the overall sensory content of a text (Paivio 1968). We extracted valence and concreteness scores for each narrative by computing mean scores of the two variables of all the words present in the text. Higher valence values indicate more positive sentiment; therefore, the final score was reversed. 

### Power Analysis

An a priori power analysis was conducted using G*Power version 3.1.9.6 (Faul et al., 2009) to estimate the sample size required to detect a medium-sized effect of trauma memory characteristics on PTSD, while adjusting for three covariates (age, sex, and narrative length) in a multiple regression framework. The analysis determined that a minimum sample size of 99 was required to detect an effect size of ² = 0.15, with 90% power, at an alpha level of 0.05.

### Data Analysis

All analyses were conducted separately for the South Africa and UK cohorts to evaluate cross-contextual generalisability while preserving site-specific variability in narrative and clinical characteristics. Prior to modelling, data were examined for outliers, distributional assumptions, missingness, and multicollinearity. Missing data on questionnaire items were minimal (< 10%). When fewer than 20% of items within a scale were missing, mean substitution within that participant’s completed items was applied to compute prorated total scores. All analyses relied exclusively on robust linear regression models to mitigate the impact of heteroskedasticity and outliers.

To evaluate the validity of each feature extraction method (Aim 1), we examined whether narrative-based indices (manual coding composites, word-count-based variables, and NLP-derived measures) corresponded to self-reported trauma memory disorganisation and sensory qualities. This was tested using robust linear regression models in which each narrative feature served as a predictor of the relevant TMQQ subscale. To assess predictive utility (Aim 2), equivalent robust regression models were estimated with standardised PTSD severity scores as the dependent variable. Narrative-based indices and self-reported memory qualities were evaluated in separate models to determine their unique associations with PTSD symptom severity. To examine developmental differences (Aim 3), all predictive models were repeated following a priori age-based stratification (< 12 years vs. ≥12 years), reflecting key developmental transitions in autobiographical memory and language ability. Differences in effect patterns were evaluated descriptively due to subgroup sample size constraints. Age and narrative length were included as covariates in all models. All analyses were conducted using R 4.3.1 (R Core Team, 2023).

## Results

### Descriptive Statistics

Descriptive statistics for demographic characteristics, narrative features, and trauma memory variables for each sample are presented in Table 1. Corresponding descriptive statistics stratified by age group (< 12 vs. ≥ 12 years) are provided in Supplementary Tables S1–S2. As shown in Table 1, participants in the UK sample produced longer narratives on average than those in the South Africa sample, whereas mean self-reported memory disorganisation and sensory scores were higher in the UK sample. Correlations between trauma memory measures, narrative length, and age are in the Supplementary Tables S3 and S4.

**Table 1:** Descriptive statistics for demographics, word count, narrative-derived features, and self-reported trauma memory scores.

**Variables**	South Africa			UK		
	M	SD	Range	M	SD	Range
Age	12.55	2.53	8.04–16.95	10.91	2.77	6–17.75
Children	9.97	1.15	8.04–11.92	9.78	1.85	6–12
Adolescents	14.38	1.39	12.05–16.95	14.8	1.66	12.04–17.75
PTSD scores	0.32	0.16	0–1	0.28	0.23	0–1
Narrative word count	263.48	297.21	19–2262	422.79	351.86	26–1841
Manual coding						
Disorganisation features*	2.12	2.34	-4.84–14.48	7.45	2.47	-1.08–16.42
Overall disorganisation	*4.29*	1.29	0–10	4.46	2.33	0–10
Sensory features*	4.28	1.01	-0.56–5.38	6.17	3.12	-1.43–15.22
Word-count based						
Disorganisation	63.24	18.36	0–86.46	70.94	17.44	0–102.18
Sensory features	3.05	1.90	0–12.95	2.75	1.71	0–15.56
NLP-derived						
Coherence	0.59	0.09	0.40–0.95	0.64	0.11	0.34–0.92
Sensory features	2.64	0.12	2.35–3.04	2.48	0.10	2.02–2.84
Self-reported						
Disorganisation	1.58	0.59	1–4	2.25	0.85	1–4
Sensory features	1.65	0.65	1–4	2.12	0.68	1–3.89

*Note: The `Disorganisation features’ and ‘Sensory features’ variables are determined by summing the z-scores across coding categories

### Validity of Narrative Feature Extraction Methods

Across both samples, narrative-based measures showed minimal evidence of convergent validity. In the South African sample (*N* = 222; df = 218), none of the manual, word-count, or NLP indicators significantly predicted either trauma memory disorganisation or sensory qualities (Table 2). A comparable pattern emerged in the UK sample (*N* = 185; df = 181), with the exception that the LIWC sensory score showed a weak but statistically significant association with self-reported sensory qualities (*β* = 0.14, SE = 0.05, *t* = 3.14, *p = .*002, f^2^ = 0.05; Table 3). According to Cohen’s (1988) benchmarks, this represents a small effect. No method demonstrated significant predictive power for self-reported disorganisation in either context.

**Table 2:** Regression summaries of narrative-derived variables predicting self-reported memory characteristics in the South African sample

Models, predictors	Self-reported sensory qualities	Self-reported disorganisation
Manual coding		Disorganisation features
Memory characteristic	β = –0.22, SE = 0.33, t = − 0.67, *p* = .504, f^2^ < 0.01	β = –0.07, SE = 0.06, t = − 1.20, *p* = .230, f^2^ < 0.01
Age	β = 0.06, SE = 0.02, t = 2.93, *p* = .004**, f^2^ = 0.04	β = –0.03, SE = 0.02, t = − 1.38, *p* = .171, f^2^ < 0.01
Narrative length	β ≈ 0.00, SE = 0.01, t = 1.19, *p* = .235, f^2^ < 0.01	β ≈ 0.00, SE = 0.00, t = − 1.30, *p* = .196, f^2^ < 0.01
		Overall disorganisation score
Memory characteristic	–	β = 0.04, SE = 0.11, t = 0.37, *p* = .320, f^2^ < 0.01
Age	–	β = –0.12, SE = 0.12, t = − 1.02, *p* = .267, f^2^ < 0.01
Narrative length	–	β = 0.02, SE = 0.02, t = − 1.03, *p* = .196, f^2^ = 0.01
Word count approach		
Memory characteristic	β = –0.04, SE = 0.04, t = − 1.08, *p* = .259, f^2^ = 0.01	β = –0.03, SE = 0.05, t = 0.61, *p* = .541, f^2^ < 0.01
Age	β = –0.06, SE = 0.02, t = 2.64, *p* = .005**, f^2^ = 0.03	β = –0.04, SE = 0.03, t = 1.16, *p* = .053, f^2^ = 0.01
Narrative length	β ≈ 0.00, SE = –0.01, t = 0.08, *p* = .976, f^2^ < 0.01	β ≈ 0.00, SE = 0.01, t = 1.68, *p* = .467, f^2^ = 0.01
NLP approach		
Memory characteristic	β = 0.02, SE = 0.01, t = 4.12, *p* = .906, f^2^ < 0.01	β = –0.35, SE = 0.53, t = − 0.66, *p* = .906, f^2^ < 0.01
Age	β = 0.01, SE = 0.00, t = 1.67, *p* = .788, f^2^ = 0.01	β = 0.054, SE = 0.02, t = 2.79, *p* = .788, f^2^ < 0.01
Narrative length	β ≈ 0.00, SE = 0.01, t = − 1.61, *p* = .108, f^2^ = 0.01	β ≈ 0.00, SE = 0.00, t = − 1.61, *p* = .548, f^2^ < 0.01

*p<.05; **p<.01; ***p<.001

**Table 3:** Regression summaries of narrative-derived variables predicting self-reported memory characteristics in the UK sample

Models, predictors		Self-reported sensory qualities	Self-reported disorganisation
Manual coding			Disorganisation features
Memory characteristic	β = 0.50, SE = 0.31, t = 1.62, *p* = .107, f^2^ =0.01		β = 0.27, SE = 1.08, t = − 0.25, *p* = .092, f^2^ < 0.01
Age	β = –0.01, SE = 0.02, t = − 0.37, *p* = .710, f^2^ < 0.01		β = –0.06, SE = 0.04, t = − 1.49, *p* = .137, f^2^ = 0.01
Narrative length	β ≈ 0.00, SE = 0.00, t = 1.07, *p* = .286, f^2^ < 0.01		β ≈ 0.00, SE = 0.00, t = − 0.005, *p* = .770, f^2^ < 0.01
			Overall disorganisation score
Memory characteristic	—		β = 0.03, SE = 0.04, t = 0.73, *p* = .464, f^2^ < 0.01
Age	—		β ≈ 0.00, SE = 0.01, t = − 1.02, *p* = .492, f^2^ < 0.01
Narrative length	—		β = –0.02, SE = 0.02, t = − 1.03, *p* = .494, f^2^ < 0.01
Word count approach			
Memory characteristic	β = 0.14, SE = 0.04, t = 3.14, *p* = .002**, f^2^ = 0.05		β = –0.02, SE = 0.03, t = − 0.75, *p* = .130, f^2^ < 0.01
Age	β = –0.01, SE = 0.02, t = − 0.41, *p* = .680, f^2^ < 0.01		β = –0.00, SE = 0.00, t = − 0.79, *p* = .948, f^2^ < 0.01
Narrative length	β ≈ 0.00, SE = –0.01, t = 0.49, *p* = .619, f^2^ < 0.01		β ≈ 0.00, SE = 0.01, t = − 0.85, *p* = .554, f^2^ < 0.01
NLP approach			
Memory characteristic	β = 0.59, SE = 0.66, t = 0.89, *p* = .543, f^2^ < 0.01		β = 0.93, SE = 1.15, t = 0.81, *p* = .893, f^2^ < 0.01
Age	β = 0.12, SE = 0.20, t = 0.61, *p* = .591, f^2^ < 0.01		β = –0.03, SE = 0.02, t = − 1.73, *p* = .234, f^2^ = 0.02
Narrative length	β ≈ 0.00, SE = 0.01, t = 0.82, *p* = .412, f^2^ < 0.01		β ≈ 0.00, SE = –0.01, t = 0.73, *p* = .398, f^2^ < 0.01

*p < .05; **p < .01; ***p < .001

### Prediction of Post-Traumatic Stress Symptom Severity

**Fig. 1:**
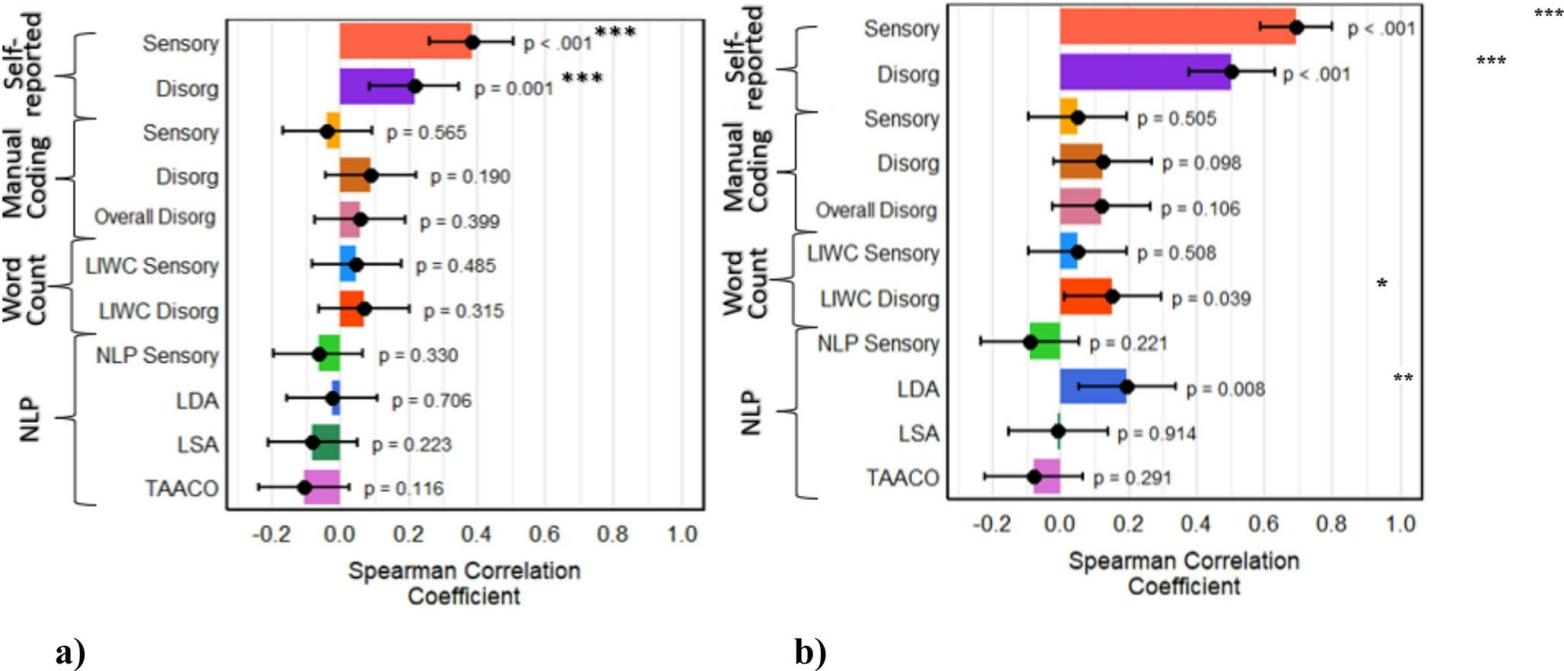
Bar graphs showing correlations between feature extraction methods and PTSD severity, in the (**a**) South African and (**b**) UK samples

The second aim of this study was to assess the extent to which memory characteristics derived from each method predict the severity of PTSD symptoms. Robust regressions were estimated with PTSD severity as the dependent variable. Narrative-based measures did not significantly predict PTSD scores in either the South African or UK samples (Table 4). In contrast, self-reported trauma memory qualities consistently predicted PTSD scores. In the South African sample, both sensory qualities (*β* = 0.35, SE = 0.05, *t* = 7.64, *p* < *.*001, f^2^ = 0.27) and disorganisation (*β* = 0.22, SE = 0.07, *t* = 3.11, *p = .*002, f^2^ = 0.04) were significant positive predictors. These represent medium-to-large and small effect sizes, respectively (Cohen, 1988). A similar pattern was observed in the UK sample, where sensory qualities (*β* = 0.37, SE = 0.06, *t* = 6.64, *p* < *.*001, f^2^ = 0.24) and disorganisation (*β* = 0.29, SE = 0.06, *t* = 4.52, *p* < .001, f^2^ = 0.11) showed robust associations with PTSD scores, reflecting medium effect sizes (Cohen, 1988). Thus, only self-report measures demonstrated meaningful predictive utility across cultural contexts. The correlations between memory characteristics derived from each method and PTSD severity are presented in Fig. 1 for both the South African and UK samples.

**Table 4:** Regression summaries for trauma memory characteristics predicting PTSD severity

Models, Predictors	South Africa	UK
Manual coding		
Sensory	β = –0.26, SE = 0.42, t = − 0.62, *p* = .536, f^2^ < 0.01	β = 0.17, SE = 0.12, t = 1.43, *p* = .154, f^2^ = 0.01
Disorganisation	β = 0.62, SE = 0.37, t = 1.67, *p* = .096, f^2^ = 0.01	β = 0.06, SE = 0.05, t = 1.22, *p* = .224, f^2^ < 0.01
Overall disorganisation score	β = 0.32, SE = 0.24, t = 1.32, *p* = .073, f^2^ = 0.01	β = 0.12, SE = 0.12, t = 0.99, *p* = .492, f^2^ < 0.01
LIWC		
Sensory	β = 0.54, SE = 0.53, t = 1.01, *p* = .492, f^2^ < 0.01	β = 0.00, SE = –, t = 1.17, *p* = .137, f^2^ = 0.01
Disorganisation	β = 0.01, SE = 0.01, t = 0.91, *p* = .587, f^2^ < 0.01	β = 0.28, SE = 0.28, t = 1.01, *p* = .126, f^2^ = 0.01
NLP		
Sensory	*β* = 0.58, SE = 0.41, *t* = 1.98, *p = .*059, f^2^ = 0.02	*β* = 0.12, SE = 0.13, *t* = -1.98, *p = .*491, f^2^ = 0.02
Coherence	*β* = − 0.11, SE = − 0.12, *t* = -2.06, *p = .*312, f^2^ = 0.02	*β* = − 0.17, SE = 0.11, *t* = -2.47, *p = .*175, f^2^ = 0.03
Self-reported		
Sensory	β = 0.35, SE = 0.05, t = 7.64, *p* < .001***, f^2^ = 0.27	β = 0.37, SE = 0.06, t = 6.64, *p* < .001***, f^2^ = 0.24
Disorganisation	β = 0.22, SE = 0.07, t = 3.11, *p* = .002**, f^2^ = 0.04	β = 0.29, SE = 0.06, t = 4.52, *p* < .001***, f^2^ = 0.11

*p < .05; **p < .01; ***p < .001

### Developmental Differences

The third aim of this study was to examine whether the utility of each memory characteristic extraction methodology for predicting PTSD differs between children and adolescents. To assess age-related differences, analyses were repeated separately for younger (< 12 years) and older (≥ 12 years) participants. None of the narrative-based approaches significantly predicted PTSD in either age group in the South African or UK sample (see Supplementary Tables S4 and S5). 

**Fig. 2:**
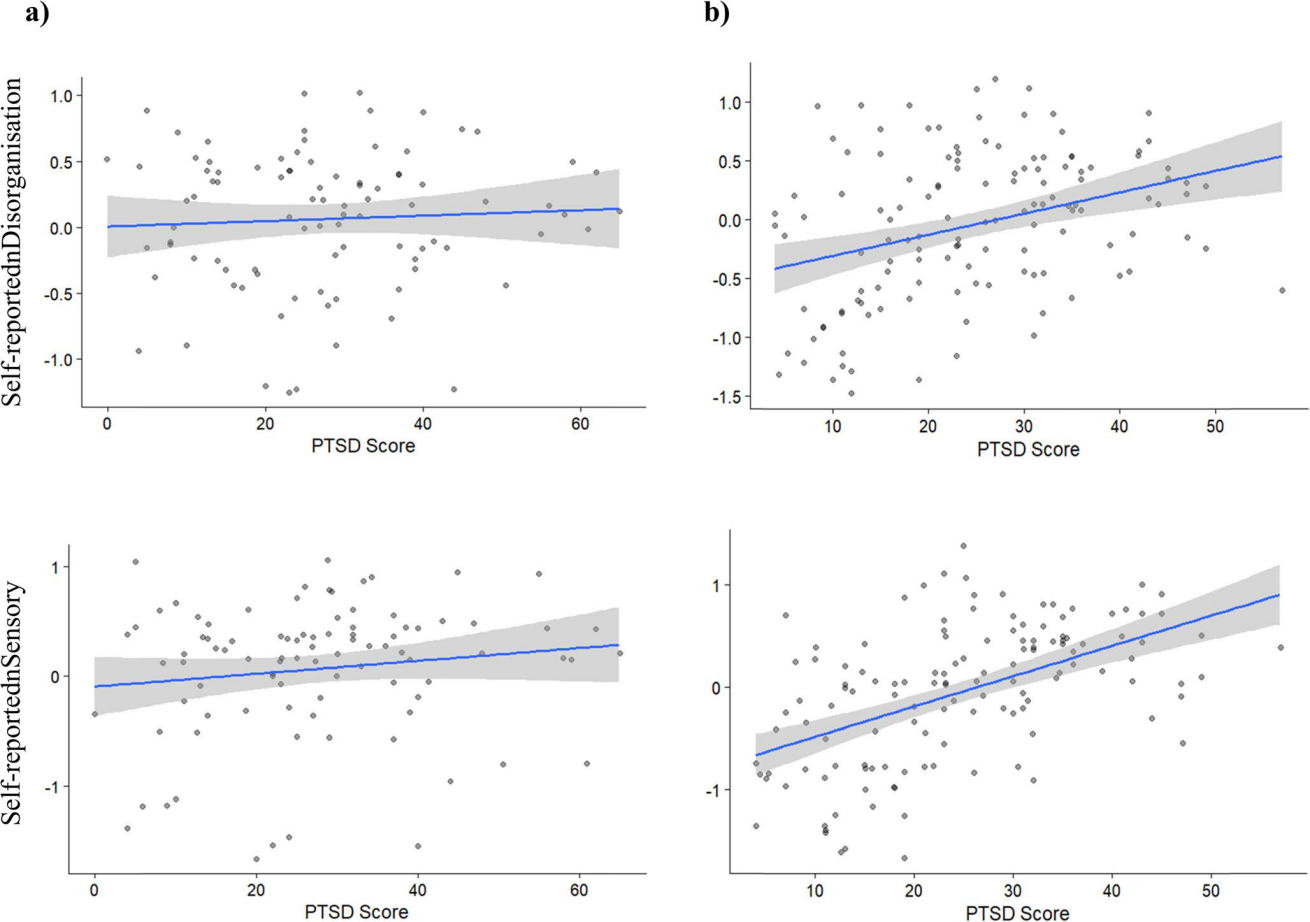
Regression plots of self-reported trauma quality scores and PTSD scores in children younger than 12 years old (**a**) and adolescents older than 12 years old (**b**) in the South African sample

In the South African sample of younger children (*N* = 92; df = 88) aged 8.04–11.92 (M = 9.97, SD = 1.15), neither the sensory nor the disorganisation subscales of the TMQQ significantly predicted PTSD severity scores (respectively, *β* = 0.14, SE = 0.33, *t* = 0.42, *p = .*205, f^2^ < 0.01 and *β* = 0.05, SE = 0.12, *t* = 0.42, *p = .*675, f^2^ < 0.01). However, among adolescents (*N* = 130; df = 126) aged 12.05–16.95 (M = 14.38, SD = 1.39), both the sensory and disorganisation subscales were found to predict PTSD (respectively, *β* = 0.52, SE = 0.08, *t* = 6.26, *p* < *.*001, f^2^ = 0.31 and *β* = 0.34, SE = 0.08, *t* = 4.09, *p* < .001, f^2^ = 0.13), representing large and medium effect sizes, respectively (f^2^ > 0.15; Cohen, 1988). Graphical depictions of the estimated robust linear regression model are shown in Fig. 2a and b for children and adolescents, respectively.

**Fig. 3:**
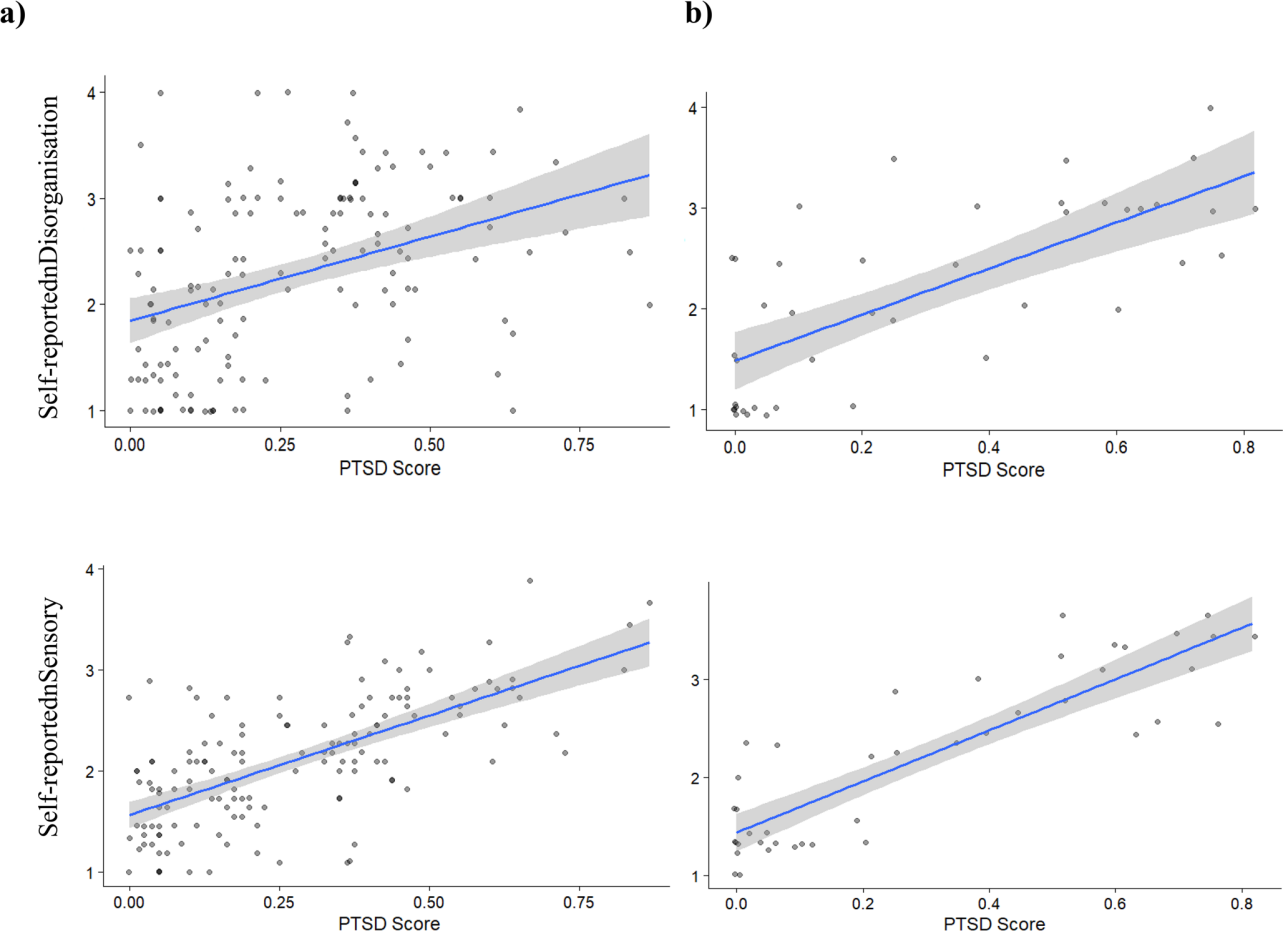
Regression plots of self-reported trauma quality scores and PTSD scores in children younger than 12 years old (**a**) and adolescents older than 12 years old (**b**) in the UK sample

In the UK sample of younger children (*N* = 143; df = 139) aged 6–12 (M = 9.78, SD = 1.85), both the sensory and disorganisation subscales of the TMQQ significantly predicted PTSD severity scores (respectively, *β* = 0.23, SE = 0.04, *t* = 6.45, *p < .*001, f^2^ = 0.30 and *β* = 0.03, SE = 0.002, *t* = 3.98, *p* < .001, f^2^ = 0.11). Equally, for adolescents (*N* = 42; df = 38) aged 12.04–17.75 (M = 14.79, SD = 1.66) in this sample, both the sensory and disorganisation subscales were found to predict PTSD (respectively, *β* = 0.28, SE = 0.06, *t* = 4.50, *p* < *.*001, f^2^ = 0.53 and *β* = 0.23, SE = 0.05, *t* = 3.94, *p* < *.*001, f^2^ = 0.41). Graphical depictions of the estimated robust linear regression model are shown in Fig. 3a and b for children and adolescents, respectively.

## Discussion

Across two socio-cultural contexts, three main findings emerged. First, narrative-based methods demonstrated a lack of validity, showing no convergence with self-reported trauma memory qualities and little to no predictive value for PTSD symptom severity. Second, self-reported disorganisation and sensory properties of trauma memory emerged as reliable correlates of PTSD. Third, developmental patterns differed by context, such that self-reported trauma memory qualities predicted PTSD from early childhood in the UK, but only from early adolescence onward in South Africa.

The most central and unexpected finding was the systematic failure of any narrative-based approach to show convergence with self-reported trauma memory characteristics. This finding offers important new insight. Narrative-derived features have been used in the past literature as measures of memory characteristics to test models of PTSD in both adult and youth research (Halligan et al., 2003; McGuire et al., 2021; Miragoli et al., 2019; Salmond et al., 2011), with several studies reporting null or inconsistent associations with PTSD symptoms, especially in youth. Yet, in our data, narrative-derived indices did not capture any variance in children’s subjective experience of trauma memory, indicating a fundamental lack of construct validity, at least in paediatric samples. However, it is critical to consider that these indices were largely developed and validated in English-speaking adult populations, and may not adequately capture how trauma is narrated in different linguistic and cultural contexts. In particular, narratives produced in isiXhosa and subsequently translated into English may not preserve the morphosyntactic, pragmatic, or culturally embedded features that these tools are designed to detect. In addition, all narrative-based methods failed to predict participants’ concurrent PTSD severity. Together, these findings challenge the assumption that the cognitive mechanisms outlined in cognitive PTSD models manifest reliably in narrative structure in youth. Unlike adults, children may be unable to express core memory processes into coherent verbal descriptions, due to limitations in language development, narrative organisation, and metacognitive insight (Nelson & Fivush, 2004). These developmental constraints may be further amplified in contexts where narrative elaboration and verbal emotional expression are less emphasised in everyday communication or educational settings.

To our knowledge, only one study has attempted to evaluate multiple trauma-memory measures within the same participants (Rubin et al., 2016). Although that study found no reliable differences in coherence across trauma versus non-trauma memories or between adults with high versus low PTSD symptoms, its modest sample size meant that only large effects could be detected, and its focus was limited to coherence rather than other theoretically relevant dimensions such as sensory vividness. Moreover, adult findings remain mixed overall: while some researchers report that disorganisation or fragmentation is elevated among those with PTSD (e.g., Halligan et al., 2003; Salmond et al., 2011), others have observed null associations (e.g., Jelinek et al., 2010; McGuire et al., 2021).

The present study advances this literature in several important ways. First, we broaden the conceptual scope to include both disorganisation and sensory memory vividness, a dimension central to cognitive models (e.g., Brewin, 2014). Second, we test these associations during childhood and adolescence, periods when autobiographical memory structure and narrative skills are still developing and may affect the detectability of memory-based processes. Third, we draw on substantially larger and culturally diverse samples than previous studies, including youth from a low- and middle-income country context, thereby increasing statistical power and evaluating the cross-cultural generalisability of narrative-based assumptions. Taken together, our findings extend and qualify existing adult-based conclusions: narrative-derived indicators show not only a lack of clinical utility, but also poor convergent validity with self-reported memory qualities, indicating that, in youth, they may not reliably index the cognitive-memory mechanisms they are intended to represent.

In contrast, in our study, self-reported trauma memory fragmentation and sensory qualities showed strong and consistent associations with PTSD symptoms in both countries. This aligns with prior evidence (Meiser-Stedman et al., 2019; McGuire et al., 2021) and supports the view that children’s subjective appraisal of their memory is a more proximal marker of clinical risk than objective narrative structure. However, the utility of self-report appears to depend on educational, cultural, and socio-environmental experiences. In the UK sample, self-report trauma memory characteristics were correlated to PTSD symptoms in both younger children and older adolescents, whilst in the South African sample, only adolescents above 12 years old showed this association. This pattern may reflect contextual differences in how younger children interpret and respond to introspective questionnaire items. For example, differences in educational practices, exposure to self-reflective questioning, and familiarity with verbally articulating internal states may influence children’s ability to engage with self-report measures. In line with recommendations to situate psychological findings within broader structural and historical contexts (Buchanan et al., 2021), these differences should not be interpreted as inherent group differences but rather as reflecting inequities in education, healthcare access, and trauma exposure. Specifically, variation among samples in linguistic framing, educational context, and familiarity with self-reflective reporting formats may have shaped how children in each sample appraised and reported trauma memory characteristics (Van et al., 2019). Equally, cultural norms around emotional disclosure and mental health may explain this pattern. Studies in South Africa describe pervasive stigma, limited mental-health literacy and substantial barriers to help-seeking among youth and families, including concerns about judgment, confidentiality and appropriateness of services (Mokitimi et al., 2019; Monnapula-Mazabane & Petersen, 2023; Morris et al., 2016).

Although stigma-related concerns about social evaluation typically intensify during adolescence, contemporary adolescents in urban South African contexts may also be increasingly empowered to recognise and challenge stigma, creating a more complex interaction between social expectations and disclosure. These intersecting developmental and cultural influences may discourage younger children from openly describing psychological distress or memory experience. Early PTSD risk may therefore be underestimated in such groups, underscoring the need for culturally sensitive screening tools.

### Strengths and Limitations

This current study has several strengths. By combining large-scale cohorts from two distinct sociocultural contexts, evaluating multiple trauma memory measurement approaches, and spanning both children and adolescents, the research offers novel insight into the developmental and cultural generalisability of trauma memory assessment methodologies in new demographics. However, several limitations should be noted. In the South African sample, despite rigorous translation procedures, subtle differences in meaning across translations may have affected the validity of narrative-based measures. The cross-language and cross-cultural context complicates the direct comparability of linguistic indices originally developed for English-speaking adults. Second, although the methods and procedures across the three secondary studies were closely aligned, important contextual differences remain. For instance, interpersonal violence and cumulative trauma are more prevalent in South African peri-urban low-income communities than in the UK emergency department context (Mitra et al., 2022; Seedat et al., 2004), and our focus on the index hospitalisation event under-captured the broader trauma histories of South African participants. Those who have experienced more frequent interpersonal trauma have been shown to be more at risk of impairments to autobiographical memory functioning (Borrelli et al., 2024). For these children, who may be disproportionately represented in the South African sample, unaccounted trauma experiences may have impacted trauma memory processing. Future designs would benefit from considering how previous trauma history interplays with trauma memory characteristics for indexed trauma events. Third, the design was cross-sectional with respect to our aims and the primary analyses reported here, limiting our ability to draw conclusions about the prediction of longer-term PTSD trajectories. Longitudinal work is needed to determine whether the patterns we observe extend to chronic symptoms and treatment response. Finally, we did not collect matched control narratives (e.g., non-traumatic negative or positive events) in this study, preventing us from testing whether any observed narrative features are specific to trauma memories versus autobiographical memories more broadly (Reed et al., 2024).

### Future Work

Future work should examine whether more fine-grained, locally focused narrative coding (e.g., focusing on the most emotionally intense or distressing moments within the trauma narrative), as recommended in adult work (Brewin, 2014), yields stronger links with PTSD than the global indices examined here. Second, research should prioritise the development and validation of developmentally appropriate, culturally grounded measures of trauma memory qualities for younger children in LMICs. This might include pictorial response formats, interactive digital tools, and play-based paradigms that reduce literacy demands and reliance on sophisticated metacognitive language, alongside formal tests of measurement invariance and qualitative work to ensure conceptual equivalence. This should also consider potential confounds which may be introduced through translation processes. This would determine whether the lack of validity and predictive utility of narrative-based methodologies can be attributed to population-specific factors or to translated isiXhosa. Finally, intervention studies, particularly those evaluating trauma-focused cognitive therapies in children exposed to multiple and chronic trauma, should routinely assess trauma memory qualities over time to clarify whether perceived changes in memory characteristics mediate symptom improvement, and whether such mediation differs across cultural contexts.

## Conclusions

This study evaluated multiple approaches to assessing trauma memory in children and adolescents following acute trauma, comparing self-report indices with manually and automatically coded narratives across youth in the UK and South Africa. Across methods, settings, and age groups, narrative-based indices showed minimal convergence with self-reported trauma memory qualities and failed to predict PTSD. In contrast, children’s and adolescents’ own ratings of disorganisation and sensory vividness were robustly associated with PTSD symptoms in both countries, although clinically informative from early childhood in the UK, but only from early adolescence in South Africa. These findings demonstrate that, in youth, the linguistic structure of trauma narratives is a poor proxy for the cognitive mechanisms emphasised in leading PTSD models. In contrast, subjective appraisals of trauma memories are reliable markers of clinical risk. Practically, brief self-report tools emerge as a scalable, low-burden strategy for early identification of PTSD, with particular promise for stepped-care triage. At the same time, the developmental and cross-cultural differences observed highlight the need for culturally sensitive, developmentally tailored assessment approaches in LMIC contexts. Overall, this work advances theory and practice by clarifying how trauma memory should, and should not, be measured in young people following trauma.

## Electronic Supplementary Material

Below is the link to the electronic supplementary material.

Supplementary file 1 (DOCX 43.7 KB)

## Data Availability

The data analysed in this study are not publicly available due to ethical restrictions and participant confidentiality agreements. Anonymised data may be made available from the corresponding author upon reasonable request.
