# Supplementary material for: Cross-Cultural Validation of Manual and Automated Methods for Extracting Trauma Memory Features and Predicting PTSD in Young Populations
Source: Res Child Adolesc Psychopathol. 2026 Jun 11;54(3):75. doi: 10.1007/s10802-026-01469-4 (PMC13253658; doi:10.1007/s10802-026-01469-4)
Supplement: Supplementary file 1 — (DOCX 43.7 KB) [file 10802_2026_1469_MOESM1_ESM.docx]

**Supplementary Materials**

**Table S1.** *South African sample: descriptive statistics for demographics, word count, narrative-derived features, and self-reported trauma memory scores divided by age groups.*

| **Variable** | **Children** | | | **Adolescents** | | |
| --- | --- | --- | --- | --- | --- | --- |
|  | **M** | **SD** | **Range** | **M** | **SD** | **Range** |
| ***Age*** | 9.96 | 1.15 | 8.04–11.92 | 14.38 | 1.39 | 12.05–16.95 |
| ***PTSD scores*** | 27.59 | 14.74 | 0–65 | 24.77 | 11.87 | 4–57 |
| ***Narrative word count*** | 148.69 | 130.13 | 19–808 | 344.33 | 351.25 | 42–2262 |
| ***Manual coding*** |  |  |  |  |  |  |
| *Disorganisation features** | −0.10 | 1.72 | −4.24–5.54 | 0.06 | 2.69 | −4.84–14.48 |
| *Overall disorganisation* | 4.99 | 1.13 | 2.33–8.00 | 3.80 | 1.19 | 1.33–9.00 |
| *Sensory features** | −0.33 | 0.62 | −0.54–2.35 | 0.24 | 1.15 | −0.54–4.09 |
| ***Word-count based*** |  |  |  |  |  |  |
| *Disorganisation* | 16.88 | 11.20 | 1.90–44.28 | 11.68 | 6.06 | 1.60–36.62 |
| *Sensory features* | 3.05 | 2.32 | 0–12.95 | 3.08 | 1.56 | 0–7.94 |
| ***NLP-derived*** |  |  |  |  |  |  |
| *Coherence* | 0.47 | 0.16 | 0.07–1.00 | 0.49 | 0.12 | 0.24–1.00 |
| *Sensory features* | 2.66 | 0.14 | 2.38–3.04 | 2.62 | 0.10 | 2.35–2.98 |

**Note*: The `Disorganisation features’ and ‘Sensory features’ variables are determined by calculating the z-scores of coding categories.

**Table S2.** *UK sample: descriptive statistics for demographics, word count, narrative-derived features, and self-reported trauma memory scores divided by age groups.*

| **Variable** | **Children** | | | **Adolescents** | | |
| --- | --- | --- | --- | --- | --- | --- |
|  | **M** | **SD** | **Range** | **M** | **SD** | **Range** |
| ***Age*** | 9.08 | 1.59 | 6.00–11.87 | 13.61 | 1.90 | 12.00–17.75 |
| ***PTSD scores*** | 22.11 | 16.13 | 0–58 | 16.82 | 15.32 | 0–49 |
| ***Narrative word count*** | 400.49 | 354.22 | 26–1734 | 447.71 | 347.84 | 63–1841 |
| ***Manual coding*** |  |  |  |  |  |  |
| *Disorganisation features** | 7.38 | 2.74 | −1.08–16.42 | 7.05 | 2.54 | 0.00–15.00 |
| *Overall disorganisation* | 4.96 | 2.45 | 1.00–10.00 | 3.71 | 1.91 | 1.00–10.00 |
| *Sensory features** | 0.10 | 1.14 | −0.59–7.99 | −0.15 | 0.71 | −0.59–2.94 |
| ***Word-count based*** |  |  |  |  |  |  |
| *Disorganisation* | 15.71 | 7.83 | 0–45.13 | 19.70 | 9.64 | 8.70–52.68 |
| *Sensory features* | 2.78 | 2.03 | 0–15.56 | 2.63 | 1.15 | 0.60–6.57 |
| ***NLP-derived*** |  |  |  |  |  |  |
| *Coherence* | 0.53 | 0.09 | 0.37–0.82 | 0.49 | 0.07 | 0.34–0.68 |
| *Sensory features* | 2.48 | 0.11 | 2.02–2.84 | 2.49 | 0.10 | 2.25–2.66 |

**Note*: The `Disorganisation features’ and ‘Sensory features’ variables are determined by calculating the z-scores of coding categories.

**Table S3.** *South African sample: Correlations among age, narrative length, and trauma memory characteristics. The table presents all correlation coefficients with raw p-values in brackets. Significant associations that remained robust after Benjamini–Hochberg FDR correction are highlighted in bold.*

| **Variable** | **2.** | **3.** | **4.** | **5.** | **6.** | **7.** | **8.** | **9.** |
| --- | --- | --- | --- | --- | --- | --- | --- | --- |
| **1. Age** | .**27 (.001)** | −.03 (.678) | −.45 (<.001) | **.34 (<.001)** | .05 (.499) | **−.29 (<.001)** | .04 (.559) | −.18 (.008) |
| **2. Narrative Length** | — | **.56 (<.001)** | **−.46 (<.001)** | **.42 (<.001)** | .16 (.013) | **−.21 (.001)** | .04 (.565) | −.19 (.004) |
| **3. Disorganisation features (manual)** |  | — | .00 (.957) | .15 (.019) | .09 (.172) | −.07 (.324) | .09 (.159) | −.14 (.037) |
| **4. Overall disorganisation (manual)** |  |  | — | −.33 (<.001) | −.11 (.099) | **.28 (<.001)** | .03 (.605) | .11 (.089) |
| **5. Sensory features (manual)** |  |  |  | — | .21 (.017) | **−.22 (.022)** | .00 (.977) | −.16 (.016) |
| **6. Sensory features (WC-based)** |  |  |  |  | — | −.19 (.080) | .06 (.368) | **.18 (.007)** |
| **7. Disorganisation (WC-based)** |  |  |  |  |  | — | −.01 (.863) | **.19 (.005)** |
| **8. Coherence (NLP-derived)** |  |  |  |  |  |  | — | −.07 (.314) |
| **9. Sensory features (NLP-derived)** |  |  |  |  |  |  |  | — |

**Table S4.** *UK sample: Correlations among age, narrative length, and trauma memory characteristics. The table presents all correlation coefficients with raw p-values in brackets. Significant associations that remained robust after Benjamini–Hochberg FDR correction are highlighted in bold.*

| **Variable** | **2.** | **3.** | **4.** | **5.** | **6.** | **7.** | **8.** | **9.** |
| --- | --- | --- | --- | --- | --- | --- | --- | --- |
| **1. Age** | .12 (.101) | **−.18 (.014)** | **−.43 (<.001)** | −.12 (.103) | −.05 (.510) | .23 (.034) | .10 (.165) | **−.27 (.007)** |
| **2. Narrative Length** | — | **.22 (.003)** | **−.43 (<.001)** | **.31 (<.001)** | .04 (.629) | −.02 (.745) | −.13 (.081) | −.25 (.001) |
| **3. Disorganisation features (manual)** |  | — | .12 (.110) | **.32 (<.001)** | −.03 (.727) | −.02 (.773) | −.21 (.005) | .20 (.007) |
| **4. Overall disorganisation (manual)** |  |  | — | −.18 (.012) | .08 (.276) | −.10 (.169) | −.07 (.373) | .11 (.148) |
| **5. Sensory features (manual)** |  |  |  | — | .21 (.120) | −.05 (.485) | −.18 (.017) | −.02 (.818) |
| **6. Sensory features (WC-based)** |  |  |  |  | — | −.10 (.187) | .02 (.833) | −.11 (.121) |
| **7. Disorganisation (WC-based)** |  |  |  |  |  | — | .25 (.001) | −.22 (.003) |
| **8. Sensory features (NLP-derived)** |  |  |  |  |  |  | — | .04 (.632) |
| **9. Coherence (NLP-derived)** |  |  |  |  |  |  |  | — |

**Material A – MANUAL CODING SCHEME** **USED TO SCORE NARRATIVES**

**Adapted from Foa, DiSavino & Turk (1995)**

**CHUNKING RULES**

A chunk is a clause which may include an action but which is divided such that all words pertain to one and only one thought.{“Chunks are separated by / and **‘…’** represents a pause in prose.}

Chunking is guided by, but is not limited to, locating subject-verb phrases.

1. A second chunk may be an elaboration of a previous thought.

2. Any repetition of a precious chunk is a new chunk.

3. Causal statements such as “If-then” (as well as compound causal statements), “in order to”, “because”, “but”, “and”, “so”, etc., are considered two chunks.

4. If a sentence includes several actions, each action should be chunked separately. *However, lists of nouns or adjectives are all included within a single chunk.*

5. Unfinished clauses are considered as separate thoughts and comprise one separate chunk per thought (unfinished thoughts = sentences attempted but not completed).

6. Dots represent a pause and pauses usually imply separate chunks. However dots occurring in the middle of a complete sentence or thought should be ignored.

7. “Speech fillers” such as “um”, “you know”, “I mean”, “like”, etc., are separate chunks.

8. “Self-dialogue” (what the victims says to herself or others) is chunked according to normal rules. Dialogue of other people is one single chunk.

{E.G., I said / please don’t do that / it annoys me}

**Hierarchy Scoring Key**

Content Analysis: The table below shows the coding hierarchy. Where a chunk fits more than one category, it is assigned the code highest in the hierarchy. Repetitions are coded as the highest priority and sensation are the least important. This means that if a construct could be coded as a repetition or an unfinished, then the rater would code it as a repetition.

| **Scoring Key/Utterance category** | **Symbol to use in text** |  |
| --- | --- | --- |
| 1. Repetitions | R |  |
| 2. Organised thoughts | OT |  |
| 4. Disorganized thoughts | DT |  |
| 5. Unfinished thoughts | UT |  |
| 6. Negative feelings  7. Positive feelings  8. Pain utterances  9. Sensations | NF  PF  PN  S |  |

| Coherence Analysis: **Dimension** | **Scale** |  |
| --- | --- | --- |
| 1. Halligan Rating | 0-10 |  |

| Other Information: **Dimension** | **Code in text** |  |
| --- | --- | --- |
| Beginning of Traumatic Event | BB |  |
| End of Traumatic Event | EE |  |

**1.** **Repetition (R)**

Chunks involving repetitions of a previous chunk in which no new information is given. Any repetition occurring more than five lines away is coded according to normal rule.

Chunks sharing semantic meaning are considered as repetitions, even if the wording is not exact.*

| *Box 1. Examples of repetition* | |
| --- | --- |
| (018) Then I ..kind of put my left foot out./ I didn’t. (UT) / I didn’t. (R) / I saw the bus (/)<..> my left foot out. (R) / | (004) And then my dad (UT) /my dad (R) /um  (029) Apparently, I bended. / I bended (R)/ …./ apparently I was bending (R) |
| (004) Then they (UT) /Then they strai- (UT) /They strai- (R) /once they straightened the bone | (093) /then the other stabbed me here, /after others kicked me. /They kicked me (R)/  (093) / they ran away/ they ran (R)/ |
|  | *“...and I start fighting../ I’m fighting* |

Note 1: If any new information is given then it is *not* a repetition

Note 2: Dialogue is never coded as repetition.

*Note 3: Utterances that were clearly repeated purposefully for emphasis are not coded as repetition

Such as common linguistic devices (e.g., "we stayed and stayed" “/ played and played/” (052))

*Note 4: Repeated speech fillers are not coded as repetition

*Note 5: Chunks in which a single word only is repeated are not coded as repetition (e.g. “and.../and…/um”)

However, single words count as repetition provided they are not speech fillers such as articles and conjunctions [e.g., (093) “they ran away/ they ran (R)/)”; “ (031) / and he was collecting kit, / collecting. (R)”]

*Note 6: Utterances with objectively the same meaning but differing wording should be coded as repetition. For example, "I walked out crying"..."I left crying" (S023)

| *Box 2. Non-examples of repetition* | |
| --- | --- |
| (004) I was thinking in my head ‘stop talking about it. /Stop talking about it’ (R) | (004) And then it snapped /and I heard a snap |
| (004) where I started saying /like /everything I was hiding in my mind /I kept on saying out | (026) Stayed in for another five days. /Then.(UT) / No. /Two days. /No three days. /(laughing)Three days! /Three days! / I can talk now! /Three days. |
|  | *“I said go away../ I just*  *said go away../”* |

**Thoughts**

Thoughts are chunks reflecting cognitive processing (i.e., rationalising, questioning) of events surrounding the trauma. We are interested in disorganised thoughts, non-consecutive thoughts, organised thoughts, and unfinished thoughts.

**1. *Organized thoughts* (OT)*:*** These are utterances that involve attempts to understand what is happening (e.g. chunks indicating reasoning, realization, *causal elaboration*, hypothesis-setting, decision making, or planning). Sometimes the use of words such as ‘I remember’, ‘because’, ‘cos’ are used when the young person is trying to communicate their thinking.

Including hypothesising about the reasoning for the thoughts/ behaviours of others. For example, "The problem is I... (UT)/ those who are at Makhaza./ People from thirty-four/ they don't want them." (S042)

Reasoning told to the interviewee by another person is not considered an organised thought

| *Box 3. Examples of organised thoughts* | |
| --- | --- |
| (004) I didn’t want anything to happen to my body /so I put my hands on (UT) /on the floor (OT) | (004) I was crying (NF) /but I wasn’t crying because of the pain (OT). /I was partly crying because of the pain /but because I had never had an accident in my life (OT) |
| (004) And I was /like /really relieved (PF) /because I didn’t have to be embarrassed anymore (OT) | (032) I couldn’t wear my dressing gown /cos I couldn’t have something on it /cos it would get stuck /and it would hurt like hell. (OT) |
| *I am keeping my eyes closed so I can identify him and he won’t have to kill me* | *If he comes around to the other side of the bathroom/ I’m gonna hold onto both sides of the door/ so that he can’t come in* |
| *If I let myself go numb it won’t hurt anymore* | *He closed the door/ and he already had my wallet/ so I realised I was in trouble* |
| *I remember thin/ wondering whether I could dial 999 without taking it out of my pocket /but for feeling the /the right buttons (planning)* | *I thought it was probably a/ probably and air pistol that was designed to look like a real gun /umm/ because it seemed to be slightly flimsy (reasoning)* |
| *-I was feeling angry (NF) about how unnecessary this all was (OT) and about how mean he was being (OT)* | *-I remember that I wasn’t happy that he was blaming me for being late (OT)* |
| *I can remember I was angry that I might never see my family again (OT)* | (017) .. I felt like (sighs)… I had broken my neck or something (S) /and that I was sort of afraid in a way to say (NF) /because if I did then I wouldn’t be able to do a lot of stuff which I liked (crying). (OT) / |

| *Box 4. Non-examples of organised thoughts* | |
| --- | --- |
| (004) And that made me jump /because it was really loud | (003) Coz all I can remember is coming over the /like /the first jump /cos there was /like /two |
| (004) and then I (UT) /I wanted to go on it. /And then I went on it. / | (004) and we had to go in a taxi /cos my dad’s car was parked in the school. |
| (004) and I was like ‘Eurgh!/ What’s that?’ /Cos my bone was like bent /and it was lumpy under it. | (008) The lady who hit me.. gimme a lift to school./ Cos I couldn’t ride my bike /cos it was like completely smashed./ |

**2. *Disorganized thoughts* (DT)*:*** These are utterances that involve/imply confused or disjointed thinking such as muddled thoughts, questioning, being overwhelmed, ambiguousness, expressions of uncertainty about the participant’s memory of events, etc. ***Also includes non-consecutive thoughts (jumps around and lacks temporal consistency).***

Clear contradictions show disorganised thought (e.g., “I sleep, / no I didn’t sleep”)

Recounting their own thoughts during an incident do not count as disorganised thoughts, only thoughts while telling the narrative are counted.

Rhetorical questions are not counted as disorganised thoughts (e.g., “so he’s the doctor, right?”)

| *Box 5. Examples of disorganised thoughts* | |
| --- | --- |
| (004) I was like ‘who were these people?’ (DT) | (004) I didn’t know what was happening to my arm |
| (003) I just thought ‘where am I?    (023) After they. (UT) /Was it before or after? (DT) / | (003) I don’t know really (DT).    *-I can’t believe he’s not listening to me* |
| *I’m wondering what is happening?* | *I don’t recall who was there at the time* |
| *I don’t remember doing that* | *I was confused about where I was* |
| *-I couldn’t remember (DT) hearing anything* | *-I couldn’t remember (DT) the name of the person who..* |
| *-I’m thinking why is he doing this to me? (DT) [overwhelmed]*    *-why is this happening? (DT)* | (027)- */*Then /umm.. /we just like pulled the brakes on. /We /like /was sort of sliding a bit /but then we just crashed into him. /We span him around../ and / umm /I remember/ like /having fun listening to music (DT)/and I was looking out the window. /I looked forward./ I seen the car. /Then I just seen /like.. /when I was running out across the road (DT)/and I looked if there was any cars /and there’s a man standing there /just like to let us go by /and.. I just remembered running across the road / and looking at the car /not anything else. |

| *Box 6. Non-examples of disorganised thoughts* | |
| --- | --- |
| (008) And.. I went down like a road. /I think it’s C Avenue /or something like that. / |  |

**5. *Unfinished thoughts* (UT)*:*** These are chunks that involve a sentence attempted but not completed. Unfinished thoughts are often followed by the completed idea, which is then coded accordingly.

*Note 1: Chunks which end with a partially verbalised word, and which are followed by a chunk beginning with this same word verbalised in full, in which the idea expressed in the original chunk is completed, are not coded as unfinished thoughts, as this is typically attributable to mispronunciation (e.g. “We went strai-/ Straight into the ditch”)

| *Box 7. Examples of unfinished thoughts* | |
| --- | --- |
| (004) /And she (UT) /And she said that ‘Everything’s gonna be ok’ | (004) And they gave (UT) /and then (UT) / and then they put… |
| -And then the para- (UT) /And then my dad came | -and I was like ‘Eurgh!/ What’s that?’ /Cos my bone was like bent /and it was lumpy under it. |

**Feelings and emotions**

**7**. ***Negative feeling utterances* (NF):** Negative feelings are unpleasant emotions such as humiliation, fear, shock, and dissociative experiences such as freezing.

| *Box 8. Examples of negative emotion references* | |
| --- | --- |
| (004) and I was really scared and worried. (NF) /I was crying (NF) | (007) And I had like a mental breakdown (NF) |
| (007) On the Friday it was /like /really horrible (NF) | (004) and I was /like /really embarrassed (NF) …/And it didn’t feel nice (NF) |
|  |  |
| *I am frozen with fear* | *I am scared to death* |
| *I couldn’t feel my body anymore* |  |

**8**. ***Positive feeling utterances* (PF):** Positive feelings are pleasant emotions such as joy, relief. ‘Fine’ or `okay` is not considered a positive emotion (neutral). Lack of negative emotion does not get coded as PF (e.g., ‘I wasn’t worried’).

The utterances need to contain an objective positive emotion rather than a positive change compared to a past situation (e.g., ‘I felt better’ and ‘I feel alright now’) as those are not necessarily positive.

| *Box 9. Examples of positive emotion references* | |
| --- | --- |
| (003) I was happy I was out (PF) | (004) I felt really excited (PF) |
|  |  |

***9****.* ***Pain utterances* (PN)*:*** Utterances expressing pain experienced.

| *Box 10. Examples of pain references* | |
| --- | --- |
| (007) ‘Oww!’ (PN) | (007) and it was /like /killing after the operation. (PN) |
| (007) on the floor /like groaning /cos I was really hurting**.** (PN) |  |

| *Box 11. Non-examples of pain utterances* | |
| --- | --- |
| (004) /Calpol which (UT) /which helped me with the pain relief / |  |

**Sensory/perceptual references**

**10.** ***Sensation utterances*** **(S)**: Utterances were coded as sensations when they made reference to one of the five senses.

| *Box 12. Examples of sensation utterances* | |
| --- | --- |
| (003) Then I went up /just sorta saw the sky. (S) | (004) and I heard a snap. (S) |
| (007) and it was like … black (S)    (026) So I was just looking at this leaf in the distance / and I could just see it (S) /and then everyone <..> else was just going all blurry. (S) | (004) and everything was all /like /dazy (S).    (026) /And when I woke up I saw the seat in front of me (S) /and I couldn’t really feel my leg. (S) /And I heard paramedics talking to me (S) |
|  |  |
| *I hear him coming* | *he smells so horrible* |
| *he feels heavy on me* |  |

| *Box 13. Non-examples of sensation utterances (I watched, listened out for…non-passive, purposeful actions)* | |
| --- | --- |
| (004) And then when I looked at it |  |

**Narrative organisation ratings**

**1. Halligan Disorganization rating scale (adapted from Halligan et al., 2003)**

The following scale gives a global rating for degree of disorganisation:

| 10 | Cannot remember anything of the event. |
| --- | --- |
| 9 | Very disorganised and muddled. Events described out of sequence. Account tangential or circumstantial. Or minimum recall of event. Bizarre content. |
| 8 | Can’t remember accident but can remember either side of it |
| 7 | Disorganised account. Non-sequential. Gaps in account. Unclear what is happening. Hard to follow. |
| 6 | Can follow account but obvious large gaps, generally in sequential order |
| 5 | Can follow account but obvious small gaps/or event out of context and/or important detail missing. |
| 4 | Reasonably coherent, but either some degree of uncertainty or obvious omissions, or not in clear sequence of events. |
| 3 | Benchmark score. Events described in clear temporal sequence but no elaboration with consistent detail or reflective thinking. No clear gaps. |
| 2 | Temporally sequential. Moderate amounts of clear detail pertaining to the event and/or reflective thinking. |
| 1 | Temporally sequential. High amounts of detail relevant to the event and/or reflective thinking. |

OTHER INFORMATION:

1. **Not coded**

Chunks involving responses made to direct questions posed by an interviewer during a session are not scored. These sections of answers to questions are [instead] underlined.

**2. Rules for identifying traumatic event situations**

For the purposes of the analysis each transcript is divided into sections delineating events which occurred before the trauma, events which occurred at the scene of the trauma itself, and events which occurred after the trauma.

**CODING THE BEGINNING & ENDING OF THE ACCIDENT**

**A. Beginning of Accident (BB):** The first expressed perception by the victim that something is going wrong (i.e. the conscious realisation of danger). For example, “..and I turn toward my car/ and this man suddenly appears/he jumps out/and the first thing I notice is how tall he is.” The first perception of threat in this case is the sight of a strange man jumping out at the woman. This chunk would be coded as the beginning of the trauma.

**B. End of Accident (EE):** The first expressed perception by the victim of the end of the immediate threat. For example “I run to the elevator/and I get in/and I’m up on the lobby/ and I come out/ and I’m running towards the../the guard station”. The first perception of safety in this case is the sight of the guard station. The last chunk would be coded as the end of the trauma.
